# Eternal sunshine of the spotless cycle

**DOI:** 10.15252/msb.20198864

**Published:** 2019-04-05

**Authors:** Lendert Gelens, Silvia DM Santos

**Affiliations:** ^1^ Laboratory of Dynamics in Biological Systems, Department of Cellular and Molecular Medicine University of Leuven Leuven Belgium; ^2^ Quantitative Cell Biology Laboratory The Francis Crick Institute London UK

**Keywords:** Cell Cycle, Quantitative Biology & Dynamical Systems

## Abstract

Understanding the quantitative principles underlying the durations of each of the four cell cycle phases has remained a challenge, despite the extensive knowledge on the molecular components and mechanisms related to cell cycle control. In their recent study, Purvis and colleagues (Chao *et al*, 2019) quantify cell cycle phase durations in human cells and propose a model whereby cell cycle progression in single cells is a succession of uncoupled, memoryless phases, each composed of a characteristic rate and number of steps.

The life cycle of a cell is an orderly sequence of events whereby one cell gives rise to two daughter cells. It is controlled by a complex network of interacting genes and proteins. This dynamical and nonlinear network regulates the activity of cyclin‐dependent kinases and their opposing phosphatases, and as such, it drives the cell cycle forward through its four main phases: G1, S, G2, and M phase (Fig 1A). In somatic cells, progression through the cell cycle has been proposed to resemble falling dominoes: Each phase is a biochemical process that needs to be fully completed before the next cell cycle phase is started (Murray & Kirschner, 1989).

**Figure 1:**
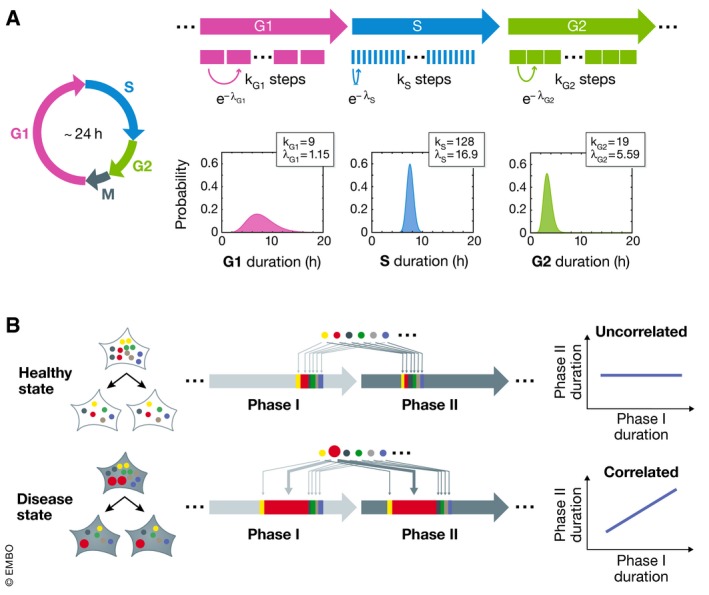
Toy model proposing uncoupling between cell cycle phase durations in health and disease states (A) The human cell cycle consists of four phases: G1, S, G2, and M phase. Each phase duration is well fitted by an Erlang distribution with a characteristic rate (λ) and number of steps (k) (λ and k values are shown for RPE cells, from Chao et al, 2019). (B) The proposed model for uncoupling of cell cycle phase durations Top: In healthy cellular states, each cell cycle phase is regulated by a large number of factors, some of them heritable, that individually exert only a minor influence on the rate of cell cycle progression. The strength exerted by an individual factor is indicated by the width of each colored box. Duration of two consecutive cell cycle phases is unlikely to be coupled in single cells. Bottom: In disease states, or upon strong perturbations, altered expression or activity of a factor that may help regulate two consecutive phases will unbalance weak influences and exert stronger control. This may result in coupling between sequential cell cycle phases.

What are the control principles that govern cell cycle progression (i.e., when is a domino pushed over and how long does it take for it to knock over the next one)? The large body of work tackling this question has shown that the answer is not a straightforward one. This is partly due to the fact that the exact cell cycle biochemical regulatory system remains incompletely understood, new interactions are still being discovered, and the rates of known reactions are often not characterized. But perhaps more importantly, cell cycle dynamics seem to be highly variable in individual cells making population studies inadequate for a quantitative understanding of cell cycle progression. Variability in protein concentration due to stochasticity in gene expression, protein partitioning during cell division, stress signals, coupling to the circadian clock, and environmental changes have all been implicated in regulating the inherent variability in cell cycle phase durations in individual cells. On the other hand, recent work (Arora *et al*, 2017; Yang *et al*, 2017) has also shown that heritable factors from mother to daughter cells enable similarities in cell cycle phase dynamics between sister cells across cell lineages.

The complexity of these regulatory events raises questions about what the principles are that underlie cell cycle phase durations, and how to reconcile the empirical observations that duration of cell cycle phases are both inherited from mother to daughter cell, yet seemingly independent in single cells.

In a recent study, Chao *et al* (2019) use quantitative measurements of cell cycle dynamics in human cells to propose a theoretical model whereby cell cycle progression in single cells is a succession of uncoupled, memoryless phases, each composed of a characteristic rate and number of steps.

The authors note that the durations of G1, S, and G2 phases are each well captured by a so‐called Erlang distribution, which describes a process that consists of a cascade of *k* independent reactions that occur at a rate λ (Fig 1A). The durations of G1 and G2 phases were found to have a wider distribution consistent with a small amount of slower reactions, while S phase duration is characterized by a narrow distribution consistent with a large amount of faster reactions. This is in agreement with previous work (Golubev, 2016).

Measuring cell cycle dynamics in three different human cell types: a non‐transformed cell line (hTERT RPE‐1), a transformed osteosarcoma cell line (U2OS), and an embryonic stem cell line (H9), the authors explored whether the duration of different cell cycle phases was correlated within individual cells ‐ in other words whether cells with a particularly slow G1 would also have a slow S phase. Surprisingly, in unperturbed conditions, no such correlation was found. This observation suggests that each cell cycle phase functions as a memoryless step independent from previous phase durations. To explain these observations, Chao *et al* proposed a mathematical toy model based on the idea that each cell cycle phase is driven by a multistep biochemical process described by the Erlang distribution. The model assumes that there is a large number of factors regulating different cell cycle durations that individually only exert a weak influence in each phase. This uncouples phase durations and their overall correlations are effectively lost (Fig 1B).

But is this always the case? The authors suggest that perhaps not. Large perturbations to shared regulators can induce these factors to become dominant and might help couple sequential cell cycle phases, inducing correlations between cell cycle phases. Chao *et al* challenged the model by perturbing cyclin‐dependent kinase 2, CDK2, an important regulator of both G1 and S phases of the cell cycle. Perturbing this shared, single factor induced coupling between cell cycle phases. This might be very relevant in the context of disease states where mutations alter expression and/or activities of key regulators and thereby affect cell cycle progression (Fig 1B). Whether cell cycle phase coupling might be a feature of cancer cells remains an intriguing hypothesis.

The study by Chao *et al* underscores the importance of integrating single cell, real‐time approaches using biosensors with mathematical frameworks for a quantitative understanding of cell cycle dynamics. We would further argue that interpretation of rich single cell data sets benefits from performing experiments with high temporal resolution (to even out technical noise) and from comprehensive theoretical analyses zooming in on sub‐populations (to explore the heterogeneity within cell populations). In future work, it will be interesting to further explore the proposed idea where each cell cycle phase is regulated by multiple factors exerting little influence on their own. Could such regulation provide tunability and robustness to cell cycle progression in healthy cells, and how might it be compromised in disease states? From a theoretical perspective, it would be worthwhile to explore whether similar decoupling of the cell cycle phases is obtained when considering nonlinear biochemical interactions. Indeed, previous work has shown that multi‐site phosphorylation/de‐phosphorylation and interlinked feedback and feedforward loops are common themes in cell cycle regulation (Ferrell & Ha, 2014). These often give rise to nonlinear dynamics and bistability (Verdugo *et al*, 2013). Bistable, irreversible switches are able to insulate seemingly connected pathways and network motifs and as such give rise to temporally decoupled cell cycle phases (Araujo *et al*, 2016; Atay *et al*, 2016). It remains to be seen to which extent these different mechanisms are complementary to explain cell cycle phase durations and control of cell cycle dynamics.
